# Response to Mechanical Stress Is Mediated by the TRPA Channel Painless in the *Drosophila* Heart

**DOI:** 10.1371/journal.pgen.1001088

**Published:** 2010-09-02

**Authors:** Sébastien Sénatore, Vatrapu Rami Reddy, Michel Sémériva, Laurent Perrin, Nathalie Lalevée

**Affiliations:** Institut de Biologie du développement de Marseille-Luminy, UMR-CNRS 6216, Université de la Méditerranée, Marseille, France; Stanford University School of Medicine, United States of America

## Abstract

Mechanotransduction modulates cellular functions as diverse as migration, proliferation, differentiation, and apoptosis. It is crucial for organ development and homeostasis and leads to pathologies when defective. However, despite considerable efforts made in the past, the molecular basis of mechanotransduction remains poorly understood. Here, we have investigated the genetic basis of mechanotransduction in *Drosophila*. We show that the fly heart senses and responds to mechanical forces by regulating cardiac activity. In particular, pauses in heart activity are observed under acute mechanical constraints *in vivo*. We further confirm by a variety of *in situ* tests that these cardiac arrests constitute the biological force-induced response. In order to identify molecular components of the mechanotransduction pathway, we carried out a genetic screen based on the dependence of cardiac activity upon mechanical constraints and identified Painless, a TRPA channel. We observe a clear absence of *in vivo* cardiac arrest following inactivation of *painless* and further demonstrate that *painless* is autonomously required in the heart to mediate the response to mechanical stress. Furthermore, direct activation of Painless is sufficient to produce pauses in heartbeat, mimicking the pressure-induced response. Painless thus constitutes part of a mechanosensitive pathway that adjusts cardiac muscle activity to mechanical constraints. This constitutes the first *in vivo* demonstration that a TRPA channel can mediate cardiac mechanotransduction. Furthermore, by establishing a high-throughput system to identify the molecular players involved in mechanotransduction in the cardiovascular system, our study paves the way for understanding the mechanisms underlying a mechanotransduction pathway.

## Author Summary

Cells sense mechanical forces and design an appropriate response crucial for cell and organ shape and differentiation during development, as well as for physiological adaptation. In particular, cardiac muscle continuously adapts to the mechanical constraints generated by its own rhythmic contractile activity. Consequently, defects in mechanosensation lead to severe pathologies, including cardiomyopathies and atherosclerosis. However, despite their well recognized functional importance, the molecular mechanisms of mechanotransduction are poorly understood. Here we study the *Drosophila* heart to investigate the genetic basis of mechanotransduction. We show that the heart responds to mechanical constraints by diastolic heart arrests, and we demonstrate that this phenotype can be used to identify genes controlling this particular mechanotransduction pathway. We show that the cation channel, Painless, first identified in the pain response pathway, also plays an essential function in the mechanotransduction pathway. The model system we have developed allows, for the first time, analysis of gene function in a mechanotransduction process *in vivo*, in the presence of endogenous mechanical constraints. These results establish the basis for an in-depth characterization of mechanotransduction pathways.

## Introduction

The conversion of physical forces into biochemical information is fundamental to development and physiology. It provides a simple means by which cells and organisms can ensure structural stability, as well as a way to regulate organ physiology [1]. Mechanosensitivity modulates cellular functions as diverse as migration, proliferation, differentiation and apoptosis, and is crucial for organ development and homeostasis. Consequently, defects in mechanotransduction, often caused by mutation or misregulation of proteins that disturb cellular or extracellular mechanics, are implicated in the development of various diseases, ranging from muscular dystrophies and cardiomyopathies to cancer progression and metastasis [2].

In addition to these long term effects on development which participate in the transcriptional control of organogenesis, mechanical stimuli can also induce acute responses. An example of acute mechanosensitive response is the pain felt by punctures or pressure exerted on the skin [3]. Mechanotransduction has also a role in hearing and balance, which result from electrochemical responses to sound waves, pressure and gravity [4]. Likewise, the cardiovascular system has to continuously mount a physiological response to changes in mechanical constraints. Regulation of vascular tone and the myogenic response occur when arterial myocytes are stretched upon elevated blood pressure [5]–[7]. Cardiac myocytes are also subjected and respond to a variety of mechanical forces, including intrinsic stretch responses which occur continuously during contraction/relaxation cycles, and extrinsic forces, induced by blood flow but also by the local mechanical environment [8].

In spite of the well recognized functional importance of mechanical constraints, the molecular mechanisms of mechanotransduction are poorly understood. In particular, little is known concerning the mechanisms underlying mechanotransduction in the cardiovascular system, and especially the heart. A major limitation is the difficulty of identifying the *in vivo* actors in mechanotransduction at work in the actual physiological environment of the whole organism in mammals, highlighting the need for more simple and tractable model systems to investigate these processes. Here we show that the *Drosophila* cardiovascular system can be used to investigate mechanosensation *in vivo* under physiological conditions. *Drosophila* is the most simple genetic model organism with a cardiovascular system, consisting of a single layered linear tube with a posterior myogenic and automatically beating heart [9], [10]. We show that the *Drosophila* heart senses and responds to mechanical forces by regulating cardiac activity and identify *painless*, a member of the TRPA family, as a key player in this process.

## Results

### Mechanosensitive response of the *Drosophila* cardiovascular system

**Figure 1:**
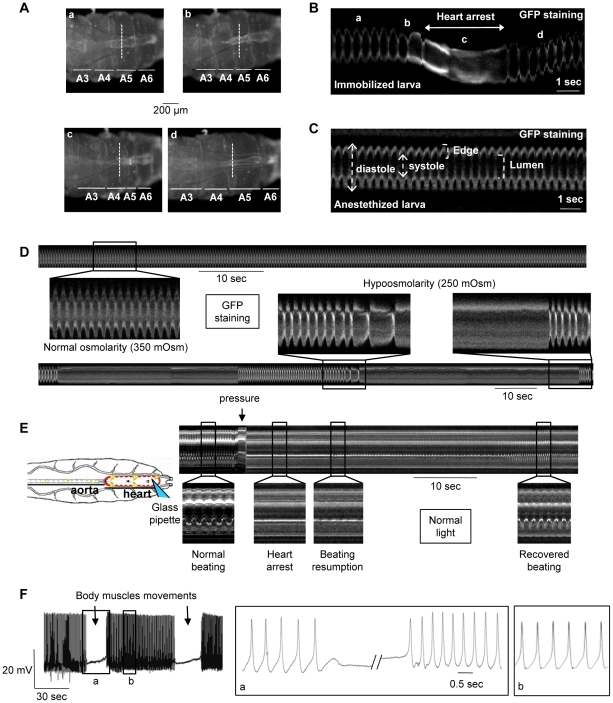
Mechanosensitive response of the cardiac tube in control (*y*,*w*; *CS* ×1029-Gal4) larvae. Heart movement detection is followed during larval motion of a larva immobilized on double-faced tape. Single movie frames (A, posterior is Right) and m-mode traces (B) are extracted from the Video S3. The m-mode trace, excised at the same position (dotted line) in all movie frames, displays a regular frequency (170 bpm) in absence of larval motion (a). When the larva attempts to crawl, the frequency decreases (b) and a 2 sec-cardiac arrest in diastole occurs during the maximal shortening of the larva (c). The basal frequency is recovered shortly following larval relaxation (d). (C) M-mode trace is extracted from the Video S2 of a typical anaesthetized larva. The cardiac tube displays a regular rhythm (170 bpm). (D) Hypoosmotic shock in semi-dissected cardiac tube. (Top) M-mode trace in normal osmolarity displays a regular rhythm (160 bpm). (Bottom) A 20% decrease in frequency is observed after 10-sec exposition to hypoosmotic solution. One minute after, periodic pauses (30–40 sec) in diastole occur after strong bradycardia and the heart restarts spontaneously at the end of the pause. (E) Direct mechanical pressure exerted on semi-dissected cardiac tube. The cardiac tube is submitted to a local pressure (posterior part of A6 segment) with a fire-polished pipette as indicated in the schematic drawing. The heartbeat is regular before the pressure, then stops when the pressure is applied and restarts slowly 15 sec after the removal of the pipette. Basal frequency is recovered after 30 sec. (F) Action Potential (AP) recording in a semi-dissected cardiac tube. The microelectrode is implanted in the posterior part of the A6 segment. Stable and reproducible APs were recorded with the characteristics previously described for *Drosophila* larvae [11]. APs stop during body muscles contraction concomitant with cardiac arrest in diastole, and a slow membrane depolarization is recorded until the depolarization phase of the next AP, when the heartbeat restarts after the wave of body muscle contraction. Note that the AP frequency is higher when the activity restarts and returns to normal frequency after 3–5 seconds. In (A–D), cardiac tubes are labeled with NP1029-Gal4-driven membrane bound GFP. In (E), heart movement detection is followed in normal light. In (D–F), preparations are bathed in Schneider medium.

In third instar *Drosophila* larvae, the heart beats with a regular frequency (around 160 bpm) [11], although interruptions are observed ranging from strong bradycardia to total cardiac arrest. These pauses, already noted by others [12], occur at particular stages of the crawling movement that accompanies the displacement of the larva on the food medium. Larval locomotion is accomplished by an accordion-like movement of the body through coordinated sequences of contraction/relaxation of body muscles [13]. To avoid larval displacement which prevents optical recording of cardiac activity, larvae were immobilized by sticking their ventral side on double-faced tape. In this condition, larvae still attempt to crawl and exhibit strong muscular contractions, but are no longer able to advance (Video S1). To monitor heart beating, m-mode traces were obtained from movies of beating larval hearts, recorded with a high-resolution video camera through the transparent cuticle, as previously described [14]. M-modes are generated by electronically excising and aligning 1-pixel-wide, vertical strips from successive movie frames (Figure 1A and 1B).

In immobilized larvae, the heart beats regularly at a constant frequency. However, when the larva moves, short pauses (from 2 to 3.5 sec, n = 10) in heart activity are observed, which coincide with a peristaltic wave of muscular contraction going through the abdominal segments (Figure 1A and 1B, Video S1). The pauses in cardiac activity, or transient cardiac arrests, appear to constitute a biological response of the heart to the mechanical constraints induced by muscular movements associated with crawling.

This hypothesis is reinforced by examining heart activity in anaesthetized larvae. In these animals, in which all body wall muscle movements are abolished, the heart beats regularly and no pauses in cardiac activity are observed (164±2 bpm, n = 160) (Figure 1C; Video S2).

### *In situ* response of the cardiac tube to mechanical force

**Figure 2:**
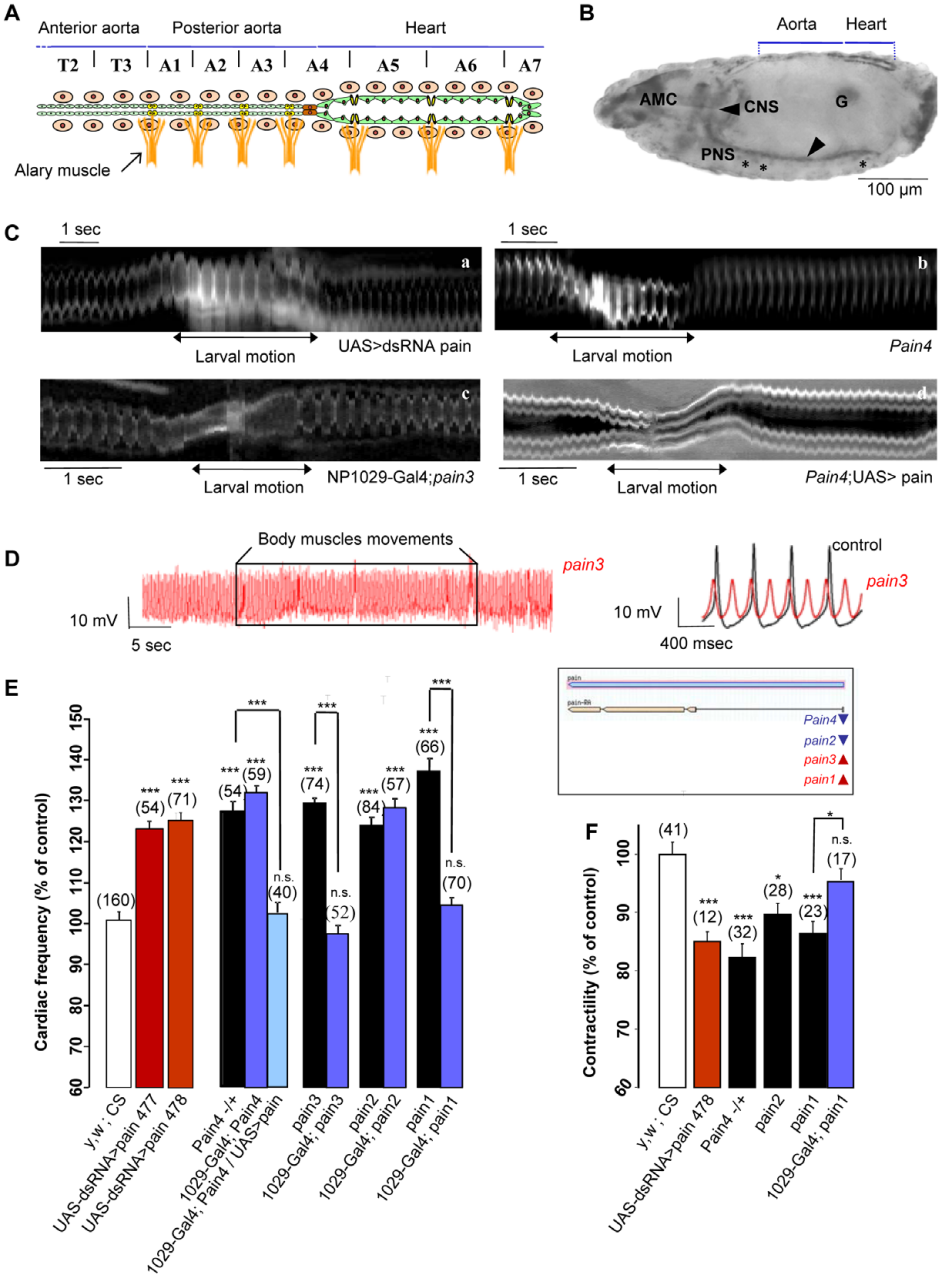
Mechanosensitive response in *pain* loss of function individuals. (A) Schema of the larval cardiovascular system. The cardiac tube is constituted by the juxtaposition of two rows of 52 cardiomyocytes (green cells) delimiting a central lumen. Metameric units, extending from thoracic segment T1 to abdominal segment A7, segment the cardiac tube. Pericardial cells (beige cells) and seven pairs of alary muscles (orange structures) are associated to the cardiac tube. (B) Lateral view of a stage 17 embryo stained with antisens *pain* RNA probe. *painless* RNA is expressed in the central (arrow) and peripheric (asterisk) nervous system, in the gonads (G), in the antennal maxillary complex (AMC) and in the cardiac tube where the expression is stronger in the heart compared to the aorta. (C) M-mode traces from movies recorded *in vivo*. Following RNAi mediated *pain* knockdown (a, extracted from Video S4) and in *pain4* mutant (b, extracted from Video S5), the heart keeps beating during larval motion with a regular frequency. This mutant phenotype is rescued by cardiac-specific re-expression of *pain* in *pain3*x1029-Gal4 (c, extracted from Video S6) and expression of the *pain* cDNA in the heart using the UAS/Gal4 system in *Pain4* (d, extracted from Video S7), in which the cardiac pause is recovered during larval motion. (D) Action Potential (AP) recording in a semi-dissected cardiac tube. (Left) Stable and reproducible APs are recorded in *pain3* mutant. Neither the frequency nor the biophysical properties were modified by body muscles contractions which were unable to stop APs. (Right) Compared to control APs, mutant APs are characterized by a slightly more positive maximum diastolic potential and reduced amplitude of depolarization, which probably leads to the observed increased frequency. (E) Cardiac frequency in RNAi lines and mutants. In *yw*, *CS*×*1029-Gal4* (control), the mean cardiac frequency was 166±2 bpm (n = 160) and represents 100% of control. RNAi mediated *pain* knockdown and the four P-element lines showed a significant increase of heart frequency. When driven by NP1029-Gal4, basal frequency is rescued by cardiac specific re-expression of *pain* in *pain1* and *pain3* but not in *pain2* and *pain4* in which the EP element is inserted in the opposite orientation and is thus unlikely to drive *pain* overexpression (see the genomic drawing of *pain* locus and mutant insertions in the insert). *Pain4* is however rescued by UAS-Painless driven by heart specific NP1029-Gal4. The mutant phenotype is also rescued by cardiac-specific expression of the *pain* cDNA in the heart using the UAS/Gal4 system in *Pain4*. (F) Contractility in RNAi lines and mutants. Contractility is measured from cardiac tube diameters as follow: (Diastole max−Systole max)/Diastole max. In *yw*, *CS*×1029-Gal4 (control), the mean contractility was 0.496±0.01 (n = 41) and represents 100% of control. In the three mutants and in the RNAi line, a significant decrease is observed following *painless* knock-down. This phenotype was fully rescued in *pain1* when crossed by the heart-specific NP1029-Gal4 line. Data are expressed as mean ± SD. *p<0.005; ** p<0.0005; ***P<0.0001; n.s.: non significative.

To further address the issue of a possible mechanosensitive response, *in situ* tests, in which mechanical forces are directly applied to the cardiovascular system, were developed. In *Drosophila* larvae, the cardiac organ is constituted by a single layered tube, so that cardiomyocytes are directly in contact with the internal (luminal) and external environments [10]. Crawling-mediated muscular movements therefore produce various changes in the mechanical forces exerted on cardiomyocytes: the sarcolemma is stretched by alary muscles contraction firmly attached to the cardiac tube (Figure 2A) and tension and shear stress modifications are induced by local changes in internal pressure and hemolymph flow. Another characteristic of the *Drosophila* heart is the circular/transversal orientation of the striated muscle fibers, running all along the heart [10], [15]. Accordingly, the above cited mechanical forces are expected to be oriented along the direction of muscle fibers. These anatomical characteristics have been taken into account for developing the *in situ* tests.

Semi-isolated cardiac tubes, prepared from ventrally open larvae (See Materials and Methods), were first subjected to a classical test of mechanosensitivity, hypoosmotic shock which induces cell swelling [16]. Consequences of cell-swelling are expected to take place at the cell membrane which is stretched by the increased volume, and at the lumen of the cardiac tube which is dramatically decreased and probably leads to an increase in intra-luminal pressure. A 24% decrease in cardiac frequency was observed after a 10-sec exposure to hypoosmotic solution (n = 11) (Table 1). This bradycardia was followed by periodic pauses in heartbeat, illustrated in Figure 1D, from 32 to 60 sec (n = 11) (Table 1). This behavior under hypoosmotic stress is consistent with the cardiac myocytes responding to mechanical stresses. Of note, similar to the observed cardiac arrest in intact larvae, hypoosmotic stress induced diastolic cardiac arrest.

**Table 1:** Averaged characteristics of cardiac defects following mechanical stimuli.

Osmotic Shock	N	Pre-stimulus Frequency (BPM)	Post-stimulus Frequency (BPM)	(Pre-Post) Frequency Variation (%)	Pause Duration (sec/5 min)	Number of Pauses (/5 min)
WT	11	159.6±2.5	121.7±3.5	−23.8	109.2±3.3	2.8±0.2
*pain1*	5	209.6±10	200±8.8	−4.6^***^	no pause	-
*pain3*	5	193.6±3.2	180±2.3	−6.7^***^	no pause	-
*Pain4*	5	196±5.1	184.8±5	−5.7^***^	no pause	-
RNAi 39477	6	187.2±6.7	172±6.5	−7.8^***^	22.5±2.7^***^	3.5±0.8^ns^
RNAi 39478	6	203±3.4	188±3	−7.3^***^	15.5±2.3^***^	4.2±0.6^ns^
1029gal4 × *pain3*	12	148.3±2.9	115.7±3.4	−22^ns/***^	97.3±4.9^ns/***^	3.6±0.4^ns/***^

Pressure Test	N	Pre-stimulus Frequency (BPM)	Post-stimulus Frequency (BPM)	(Pre-Post) Frequency Variation (%)	Pause Duration (sec/5 min)	Delay of Basal Frequency Recovery (sec)
WT	11	151.3±2.1	arrest	-	25.8±1.6	49.7±3.7
*pain3*	5	197.6±2	197.6±2	0	no pause	-
*Pain4*	5	198.4±3.7	198.4±3.7	0	no pause	-
RNAi 39477	5	178.4±1.6	arrest	-	12.8±1.7^**^	12.8±1.7^***^
RNAi 39478	5	183.2±4.3	arrest	-	9.5±0.9^***^	9.5±0.9^***^
1029gal4 × *pain3*	10	150.8±3.6	arrest	-	20.6±1.5^ns/***^	54.1±4.5^ns/***^

Frequencies, pauses and number of pauses are expressed as mean ± SD. The pause duration (in sec) corresponds to the cumulative time of pauses during 5 min. All experiments are performed in Schneider medium. The number of experiments is indicated in the Table. For 1029Gal4 × *pain3*, statistical significance is indicated on the left for data compared to wild-type and on the right when they are compared to *pain3*. In RNAi lines, observed phenotypes are weakly than in mutants probably because of a lower efficiency of RNAi in inactivating Painless.

**p<0.0005; ***p<0.0001; n.s.: non significative.

Next, a local mechanical pressure was directly applied on semi-isolated cardiac tube preparations. Cardiac myocytes of the posterior A6 segment were submitted to a pressure with a fire-polished pipette controlled by a micromanipulator. A displacement of 50 µm, from the top of the cardiomyocyte toward the central lumen, during 1 sec was found to produce an immediate 25.8±1.6 sec pause in diastole of all heart segments, followed by a gradual return to the basal frequency in 49.7±3.7 sec (n = 11) (Figure 1E and Table 1). When the pressure was maintained, the heart restarted beating after 30 sec, initially arrhythmically, subsequently recovering its basal rhythm (not shown).

This result demonstrates that direct mechanical forces exerted on cardiomyocytes induce cardiac arrest, indicating that cardiac cells are able to sense and specifically respond to mechanical stimuli.

Electrical activity of the cardiac cell is characterized by Action Potentials (APs). In the *Drosophila* heart, typical APs can be recorded with a microelectrode implanted in the contractile myocytes of the posterior heart in semi-isolated cardiac tubes [11] (Figure 1F, left panel). As previously described, *Drosophila* cardiac APs are characterized by a slow pre-depolarisation phase rather similar to sinoatrial node APs encountered in mammals [11].

In *Drosophila*, potassium and sodium concentrations in hemolymph are different from the situation in mammals. Electrochemical potentials for these two ions can be extrapolated from the measured intracellular concentrations in the moth (−32 mV for K^+^ and +22 mV for Na^+^) [17], [18] and theAP develops between these two values of potential. Importantly, this potential range corresponds to the window current of alpha 1D, the unique gene encoding L-type calcium channels in *Drosophila* [19], [20]. Since the fast sodium current is absent from *Drosophila* cardiomyocytes [21], AP depolarization is probably due to a calcium oscillation coming from alpha 1D window current.

In semi-dissected larval preparations, the cardiac tube is still attached to the dorsal epidermis and alary muscles are present. When dissection is properly performed (See Materials and Methods), the heart beats with a regular rhythm, but rare spontaneous body muscles movements were still able to promote cardiac arrests. APs exhibited a small depolarization concomitant with cardiac arrest in diastole (not shown). Then, after contraction of the body muscles concomitant to heartbeat recovery, an elevated AP frequency was observed for a few seconds before the basal frequency was recovered (Figure 1F, right panel). Interestingly, a similar increase in heartbeat rate was observed after cardiac arrest in intact immobilized larvae in which the heart stops beating in diastole during larval contraction and cardiac activity restarts with a transiently increased frequency (see above, Figure 1B; Video S1).

The combined observations made *in vivo* and in semi-dissected preparations provide strong arguments that the observed cardiac pauses constitute the physiological response to the mechanical forces exerted on the cardiovascular system.

### Screening for genes required for mechanotransduction in the *Drosophila* heart

We anticipated that the cardiac arrest phenotype might be well suited for a screen aimed at identifying genes involved in a mechanotransduction pathway. Therefore a loss of function screen was conducted using transgenic *Drosophila* lines containing inducible UAS-RNAi constructs. Genes were specifically down regulated in the cardiac tube by targeting the expression of dsRNA *via* the UAS-Gal4 system [22] with the heart-specific NP1029-Gal4 driver [15]. For most of the genes investigated, two different RNAis per gene were available or were constructed in the laboratory (see Materials and Methods).

The screen was conducted by direct observation of heart beat in non-anaesthetized, immobilized third instar larvae (Video S3). We screened for lack of pauses in heart activity or, alternatively, for bradycardia during larval contractions as they attempt to crawl on double-faced tape. Concomitantly, heartbeat parameters were also determined and analyzed on anaesthetized larvae.

A set of 17 candidate genes encoding characterized ion transporters and proteins implicated in calcium signaling pathways have been screened. These genes were selected due to the excitable and contractile nature of cardiomyocytes. The response to a mechanical signal is expected to be first converted into electrical activity that may in turn modify the intracellular calcium concentration which regulates contractility.

Results are summarized in Table S1. Knock-down of certain genes affected heart rate, including *ork1*, *SK*, *painless*; that of others, *alpha1D* and *calmodulin*, led to a complete heart arrest. However, a clear absence of cardiac pauses was only found following down regulation of *painless* (CG15860) (Video S4).

*Painless* (*pain*) encodes a member of the Transient Receptor Potential (TRP) family of cationic channels. The *pain* gene codes for a TRPA family member (Table S1), [23]), first identified as involved in noxious heat sensation [24]. Interestingly, Painless has also been proposed to mediate neuronal response to touch [24]. The protein is a heat-activated Ca^2+^-permeable channel, whose function is largely dependent on calcium [25]. Painless is expressed throughout the *Drosophila* cardiac tube [24], but we found that expression was stronger in the posterior heart than in the aorta (Figure 2A and 2B).

Identifying Painless in this screen is particularly interesting because the involvement of TRP channels in mechanosensation is under intense debate [26]. TRP channels are a large family of cation channels which play critical roles in the response to all major classes of external stimuli, including light, sound, chemicals, temperature and tension [27], [28]. TRP channels are also responsible for sensing changes in the local environment, such as alterations in osmolarity and hygrometry, and appear thus to be general factors for sensing the environment [29]. The *Drosophila* genome comprises 16 TRP channels instead of the 27–33 present in the human genome [26] among which 11 have been functionally characterized.

In mammals, several TRP subunits, including members of the TRPC, TRPV, TRPM and TRPP subfamilies, have been implicated in the force-dependent responses of the cardiovascular system [30], [31]. However, these results remain highly controversial [31], [32] mainly because of difficulties in addressing these issues in the mouse.

### *pain* function is autonomously required in the heart for the mechanosensitive response

The phenotypes observed after NP1029-Gal4 driven dsRNA mediated knockdown of *pain* were confirmed using Hand-Gal4 driver, another heart specific Gal4 driver (not shown). In addition, all four *pain* loss of function mutations previously characterized [24], reproduced the *pain* RNAi phenotypes and displayed no cardiac arrests when larvae attempt to crawl, as illustrated in Figure 2C and in Video S5. Furthermore, heart-specific expression of the *pain* cDNA using the UAS/Gal4 system was able to rescue *pain2* and *Pain4* mutant phenotypes (Figure 2C). Likewise, *pain1* and *pain3*, two EP alleles allowing *pain* overexpression when activated by a Gal4 driver ([24], see Materials and Methods and Figure 2E for a schematic description of the *pain* mutants used), were fully rescued when crossed to a heart-specific Gal4 line (Figure 2C and Video S6). By contrast, heart-specific expression of *Drosophila TRPA1*, the vertebrate homolog which has been invoked as a mechanical sensor [33]–[36], was unable to rescue the *pain* phenotype (not shown). Similarly, two other members of the TRP gene family, *nanchung* and *waterwitch*, failed to rescue the *pain* phenotype, pointing to the specificity of *pain* in the mechanosensitive response (not shown). Interestingly, in experiments where *pain* function was rescued in the heart, the *pain* mutants still exhibited a neuronal nociceptive phenotype (not shown). Together, these observations indicate that Painless is autonomously required in the heart for mechanosensation.

In addition to its function in mechanotransduction, Painless is also involved in basal cardiac activity. Heart rate measurements were performed in anaesthetized individuals to prevent any mechanical challenge coming from larval motion. *painless* knockdown led to an increased cardiac frequency from 22% in RNAi lines to 37% in mutants (Figure 2E). As observed in the case of mechanosensitive cardiac arrests, *pain* is also autonomously required in cardiac cells for its function in basal heart rate: heart specific expression of *pain* was able to rescue *Pain4* phenotypes and *pain1* and *pain3* were fully rescued when crossed by the heart-specific NP1029-Gal4 line (Figure 2E). Of note, the accelerated cardiac rate observed in *pain* mutants is correlated to increased AP frequency. AP profiles also exhibited reduced amplitude of the depolarization phase (Figure 2D, see below).

Painless has been shown to be a calcium permeable cation channel [25]. Thus, cardiomyocyte contractility was also characterized, by measuring (Diastole max – Systole max)/Diastole max diameters in three mutants and in one RNAi line. Compared to wild-type, a decrease from 10 to 18% was observed following *painless* knock-down. This phenotype was fully rescued in *pain1* when crossed by the heart-specific NP1029-Gal4 line (Figure 2F).

We then investigated *pain* function in semi-dissected cardiac tube preparations submitted to *in situ* mechanical tests. When preparing the *pain* mutant hearts, we noted their particular insensitivity. Mutant cardiac tubes, with constant and regular heart rhythm, were easily obtained, contrasting with wild-type hearts, which are extremely sensitive to all kinds of mechanical stresses arising during dissection.

**Figure 3:**
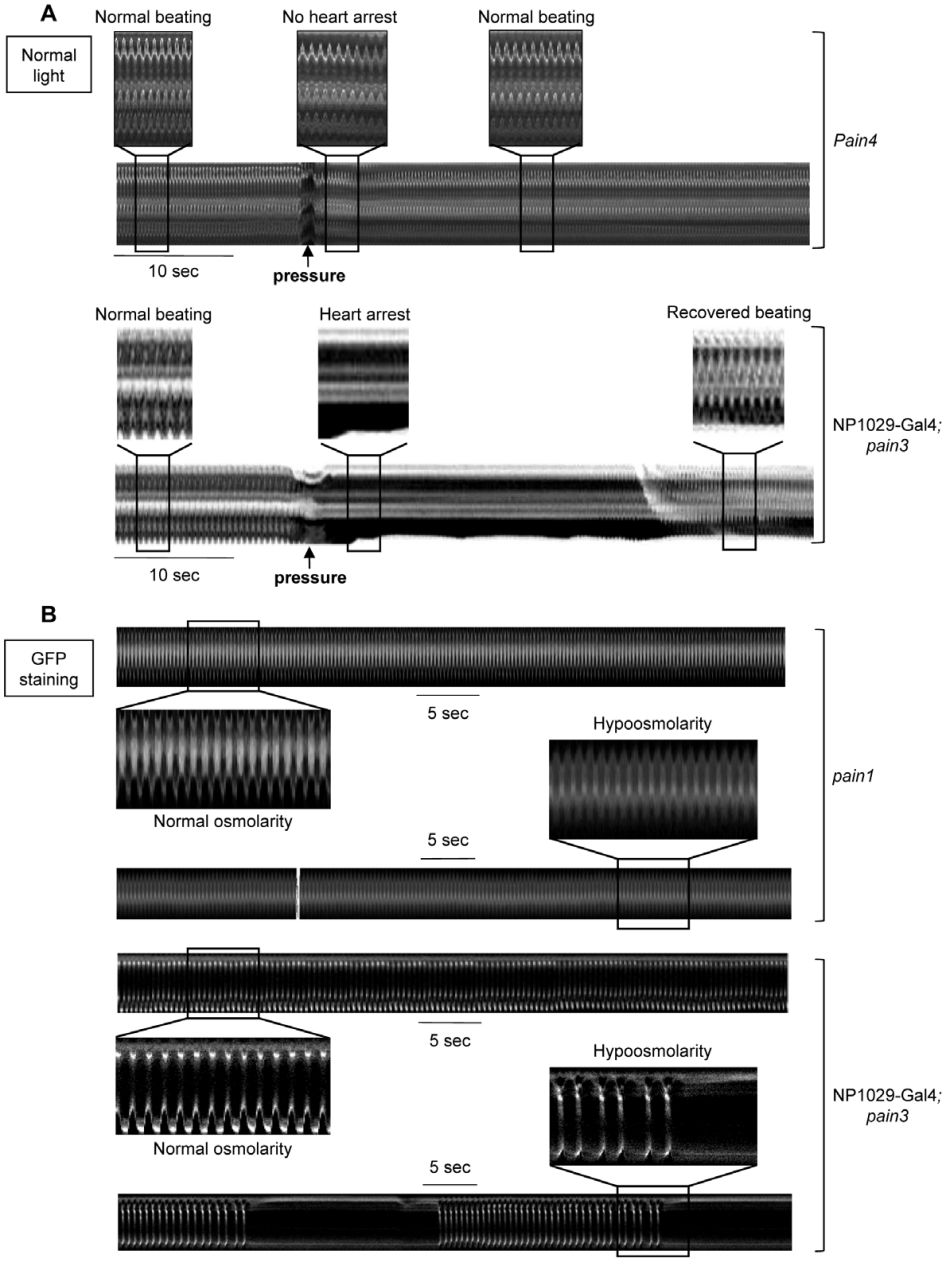
Response to *in situ* mechanical stress in *pain* knockdown individuals. (A) Direct mechanical pressure exerted on semi-dissected *pain4* cardiac tube. No modification of cardiac rhythm is observed when the mutant cardiac tube is submitted to a local pressure (applied as described for the wild-type in Figure 1E). This mutant phenotype is rescued by cardiac-specific re-expression of *pain* in *pain3*x1029-Gal4 in which the cardiac pause is recovered following the mechanical stimulus. (B) Hypoosmotic shock in semi-dissected cardiac tube. M-mode trace in *pain1* mutant displays a regular cardiac frequency in normal osmolarity (210 bpm) preserved after exposure to hypoosmotic solution. This mutant phenotype is rescued by cardiac-specific re-expression of *pain* in *pain3*x1029-Gal4 in which the periodic pauses in diastole are observed after strong bradycardia. In (A), heart movement detection is followed in normal light. In (B), cardiac tubes are labeled with NP1029-Gal4-driven membrane bound GFP. Preparations are bathed in Schneider medium.

When a local pressure was applied to segment A6, the entire cardiac tube kept beating in mutant larvae with only a very short arrest (n = 10, Table 1) precisely at the site of application (Figure 3A). Similarly, RNAi knockdown individuals displayed cardiac arrests (from 8 to 18 sec, n = 10) significantly shorter than wild-type (from 18 to 33 sec, n = 11) (Table 1). Likewise, the cardiac rhythm of *pain* mutants was not modified by hypoosmotic shock, despite visible cell-swelling (Figure 3B) and only very short cardiac pauses were observed in RNAi lines (Table 1). Conversely, re-expressing *pain* specifically in the heart restored cardiac arrests in both tests (Figure 3A and 3B and Table 1). In conclusion, mechanical stresses which led to cardiac arrests in wild-type larvae, were unable to generate cardiac pauses in *pain* mutants (compare Figure 3A and 3B to Figure 1E and 1D), indicating that *pain* is required for the mechanosensitive response.

In addition, spontaneous body muscle movements, which induced cardiac arrests and blocked APs in wild-type controls (Figure 1F), were unable to induce cardiac arrests or block APs in contractile myocytes of mutant larvae (Figure 2D). Mutant APs were characterized by an increased frequency which is probably due to a significantly reduced amplitude of depolarization and a slightly elevated maximum diastolic potential (MDP) compared to controls. This MDP value was however difficult to precisely determine with microelectrode because of the high frequency of beating leads to important background noise on the baseline. The statistical significance is, in this instance, difficult to determine. These AP characteristics are consistent with the increased heart rate observed *in vivo* in anaesthetized RNAi mediated knock-down (25%, n = 125) and mutant (from 24% to 37%, n = 278) larvae compared to wild-type (Figure 2E and Table S1).

**Figure 4:**
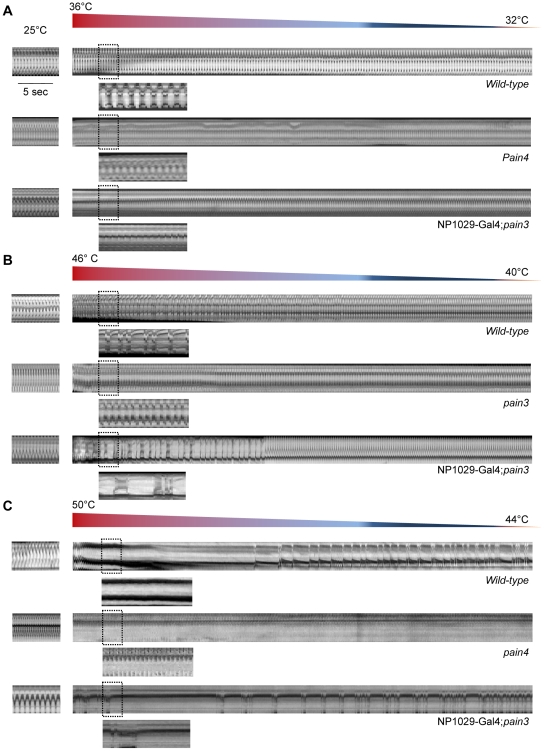
Cardiac response to Painless activation by elevated temperature. In all cases, semi-dissected preparations were first bathed in the Schneider medium at 25°C (m-modes, left). Then, the temperature of the medium in the perfusion chamber was increased at the indicated values (m-modes, right). (A) M-mode traces from movies recorded after slight temperature increase. The heart rate of wild-type cardiac tube bathed in the medium at 25°C is regular (160 bpm). When the temperature is increased to 36°C, a 14.1% increase in frequency is observed. The same type of response is observed in the *pain4* mutant (7.7%) and after transgenic rescue (12.2%). (B) M-mode traces from movies recorded after moderate temperature increase. In the wild-type, the increase of temperature from 25°C to 46°C induces a strong arrhythmia with an important decrease in the cardiac rate (−23.7%). In *pain3* individuals, the rise in temperature induces tachycardia (20.2%) but no arrhythmia was observed. The bradycardia and the associated arrhythmia observed in the wild-type are recovered in the *pain3* rescue (−18.5%). In both the wild-type and the rescue, the regular beating is progressively recovered when the temperature drops back. (C) M-mode traces from video-movies recorded during high temperature increase. In wild-type, increasing the temperature from 25°C to 50°C leads to a cardiac pause and the heart restarts to beat firstly irregularly, then the basal rhythm is recovered. In the mutant, like for both lower temperatures, increasing the temperature to 50°C induced a strong tachycardia but no cardiac arrest is visible. Rescuing *pain* expression in the heart restores the cardiac pause and the prolonged arrhythmia. The basal rate is recovered after 12 min.

Painless has been shown to be directly activated by temperature over 44°C [25]. If cardiac arrests induced by mechanical stimulation are specifically mediated by Painless, then activation of Painless by temperature should also induce cardiac arrests. We first tested the effect of temperature on heart activity. Semi-dissected cardiac tubes were bathed in medium at various temperatures, from 25 to 50°C. Wild-type larvae displayed an expected increase in heart rate from 25°C (the bath temperature in the control conditions) to 44°C. Cardiac response obtained at 36°C, 46°C and 50°C has been compared with the control conditions at 25°C. At 36°C, the heart rate was increased (14.1%, n = 11) (Figure 4A). However, at 46°C, a clear discontinuity in the temperature response was observed, with the appearance of a strong bradycardia (23.7% decrease of heart rate, n = 15) (Figure 4B and Table 2). This threshold temperature is consistent with that inducing a clear response to noxious heat touch (46°C) [24]. It is also close to the calcium gating temperature (44°C) in Painless transfected HEK139 cells [25]. Furthermore, at a temperature of 50°C diastolic pauses in heartbeat were observed (n = 10); normal heart activity was recovered when the temperature dropped (Figure 4C and Table 2).

**Table 2:** Averaged characteristics of cardiac defects following temperature shock.

Temperature Shock		N	Pre-stimulus Frequency (BPM)	Post-stimulus Frequency (BPM)	(Pre-Post) Frequency Variation (%)	Pause Duration (sec/5 min)	Delay of Basal Frequency Recovery (sec)
**36°C**	WT	11	152.4±2.7	173.8±2.7	+14.1	no pause	-
	*pain3*	5	194.4+/+5.6	207.2±3.9	+6.6^ns^	no pause	-
	*pain4*	5	198.4±3.9	213.6±5.3	+7.7^ns^	no pause	-
	RNAi 39477	11	183.6±2.3	200.7±2.3	+9.3^ns^	no pause	-
	1029gal4×*pain3*	10	148.8±2.4	166.8±2.3	+12.2^ns/ns^	no pause	-
**46°C**	WT	15	148.3±2.9	113.1±4.2	−23.7	no pause	-
	*pain3*	7	200.6±3.1	241.1±4.1	+20.2^***^	no pause	-
	*Pain4*	5	202.4±8.2	244.8±6.4	+20^***^	no pause	-
	RNAi 39477	5	182.4±4.1	215.2±2.9	+17.9^***^	no pause	-
	RNAi 39478	5	188±4	216.8±6.6	+15.3^***^	no pause	-
	1029gal4×*pain3*	10	148.6±2.4	121.2±4.3	−18.5^ns/***^	no pause	-
**50°C**	WT	10	152.8±2.2	arrest	-	26.8±2	43.8±2.4
	*pain3*	6	193.3±2.2	243.3±8	+25.9	no pause	-
	*Pain4*	5	202±2.7	257.6±5.3	+27.3	no pause	-
	RNAi 39477	5	183.2±2.3	fibrillation	-	no pause	-
	RNAi 39478	6	178.7±6	fibrillation	-	no pause	-
	1029gal4×*pain3*	10	146±3.1	arrest	-	25.5±3^ns^	42.5±2.3^ns^

Frequencies, pauses and number of pauses are expressed as mean ± SD. The pause duration (in sec) corresponds to the cumulative time of pauses during 5 min. All experiments are performed in Schneider medium. The number of experiments is indicated in the Table. For 1029Gal4×*pain3*, statistical significance is indicated on the left for data compared to wild-type and on the right when they are compared to *pain3*. In RNAi lines, observed phenotypes are weakly than in mutants probably because of a lower efficiency of RNAi in inactivating Painless. **p<0.0005; ***p<0.0001; n.s.: non significative.

In contrast, in *pain* mutants, while elevation of temperature from 25 to 44°C also induced tachycardia (7% increase of heart rate at 36°C compared to 25°C, n = 10), no bradycardia was observed at 46°C (20% increase of heart rate, n = 12), nor cardiac arrests at 50°C (26.5% increase of heart rate, n = 11), indicating that the effect of temperature observed in wild-type individuals is mediated by *pain* (Figure 4A–4C and Table 2). Finally, cardiac sensitivity to temperature over 46°C was also lost when *pain* RNAi was specifically targeted to the heart (Table 2). Moreover, cardiac expression of endogenous *pain* in *pain3* mutant larvae was sufficient to restore the temperature-dependent cardiac arrest, demonstrating the specific requirement of *pain* function in this response (n = 10) (Figure 4A–4C and Table 2).

In conclusion, as was observed after mechanical stimulation, the cardiac response to direct Painless activation by heat is a reversible cessation of heart activity. Diastolic pauses are therefore specifically induced by Painless activation in the heart, either by heat or by mechanical stimulation.

## Discussion

In this study, we provide strong evidence that the *Drosophila* cardiac tube senses mechanical constraints and responds by an adaptive cardiac response. We identified *painless*, encoding a member of the TRPA family, as an essential actor of this response.

Painless is a calcium permeable cation channel, directly activated by temperature [25]. Mechanical stimuli inducing heart pauses in wild-type larvae, like pressure and swelling, are ineffective in the absence of *pain*. Moreover, we show that Painless gating by temperature induces cardiac arrest. These data clearly show that Painless is required for the cardiac mechanical response likely mediated by elevated intracellular calcium.

Strikingly, all the stress-induced cardiac pauses are observed at the end of the relaxation phase of cardiac beating. As the activation of Painless is expected to increase the cytosolic calcium concentration, these data strongly suggest that Painless-dependent calcium influx induced by mechanical stress is not recruited by the contractile proteins to induce cardiac muscle contraction. This is reminiscent of the vasodilatation promoted by calcium uptake in vascular smooth muscles [37]. In smooth myocytes, high calcium concentration activates calcium-activated potassium channels leading to cell relaxation by hyperpolarizing the plasma membrane. However, knockdown of the two *Drosophila* calcium-activated potassium channels, Slowpoke and SK, has no effect on cardiac rhythm during crawling (see Table S1) and cardiac arrests similar to wild-type are observed in *in situ* pressure tests. These results, confirmed in the *slowpoke1* mutant (not shown) undermine the participation of calcium-activated potassium channels in this mechanism. The identification of other genes involved in the pathway will provide insight into the mechanisms by which Painless-dependent calcium influx prevents cardiomyocyte contraction.

The accelerated heart rate observed in *pain* mutants even in unchallenged anaesthetized individuals indicates that Painless is also involved in excitation/contraction coupling. Indeed, an increased cardiac frequency and less efficient contractions are observed in *painless* mutants which also exhibit a shortened depolarization phase of the action potential. This cardiac response to *pain* inactivation has to be analyzed in the light of the particular electrophysiological properties of the *Drosophila* cardiomyocyte. Because of distinct ionic concentrations in insects, potassium and sodium electrochemical potentials differ from that of mammals [18]. AP develops between −30 mV and +10 mV, in the window current for the unique voltage-activated L-type calcium channel, alpha 1D [19], [20]. Therefore, the window current leading to a constant calcium inflow is probably responsible for pacemaking. Precocious repolarization, possibly occurring because voltage-dependent potassium channels are already activated at these potentials, may increase APs frequency. This, at least in part, may explain the accelerated heart rate in absence of the depolarizing calcium current coming from Painless in unchallenged conditions. Increased cardiac frequency in *pain* mutants may also result from the observed reduced MDP even though it is difficult to establish this value. The observed depolarization at the MDP may reveal, in basal conditions, a Painless-activation of a repolarizing current at the end of the repolarization phase.

The dual role of Painless in cardiomyocytes is well illustrated by the two different outcomes observed on APs profile. Pain activity in basal cardiac function mainly takes place at the end of the depolarization phase, most probably due to calcium influx within the cell, which impacts on contraction efficiency. On the other hand, Painless activation during extrinsic mechanical stress results in the absence of fast depolarization phase. As discussed above, we hypothesize that mechanical stress-activated calcium influx will activate calcium-dependent repolarizing channels. Which mechanism(s) could account for this dual function within the same cell type? One possibility could be that Painless is localized at different sub-cellular domains within the sarcolemma. For example, localizing Painless in T-tubules might account for Painless involvement in excitation/contraction coupling, while distinct membrane localization for another Painless pool would be involved in its mechanosensitive function. Further investigations aiming at analyzing Painless sub-cellular localization in the cardiac muscle may provide clues about this dual activity as described for L-type calcium channels in mammalian cardiomyocytes [38].

In the cardiovascular system, several TRP channels have been proposed to be involved in force-dependent responses [30], [31]. However, these results remain highly controversial mainly because of difficulties in addressing these issues in the mammalian heart. *painless* codes for a TRPA family member, first identified as involved in noxious heat sensation and proposed to mediate neuronal response to touch [24]. Our data thus provide the first *in vivo* demonstration of TRPA channel function in mechanosensation in the cardiovascular system.

TRP channels seem to play a particular warning function by sensing stimuli (noxious temperatures, pungent natural compounds, environmental irritants) that can be detrimental for the organism. Blocking muscle contraction in *Drosophila* cardiomyocytes would constitute a safety function for Painless to prevent heart damage. Our findings provide direct experimental evidence that mechanical stimulation is important for heart function [39].

This paves the way for elucidating the mechanisms of an acute response to mechanical stimuli, using the fly cardiovascular system as a paradigm. Indeed, it will be possible to identify, in addition to Painless, other essential players of the mechanotransduction pathway. Of note, other TRP channels expressed in the heart are unable to compensate for loss of *pain* function, suggesting a specific “sensing” function for Painless in response to mechanical constraints in the cardiovascular system. Characterization of Painless in HEK cells failed to reveal direct activation by stretch [25], suggesting that Painless is not the direct force sensing partner of the mechanism. However, it will be particularly important to investigate this property in *Drosophila* dissociated cardiomyocytes because of the possible lack of Painless-specific interactors in HEK cells that might be critically required for mechanical force response.

Given the well acknowledged evolutionary conservation of cardiac development and function between flies and vertebrates [40]–[42], we anticipate that investigating mechanotransduction in *Drosophila* will be valuable to understand cardiac mechanotransduction mechanisms in mammals.

## Materials and Methods

### Drosophila strains

The *y*,*w*; *CS* strain, issued from a *yellow*, *white* strain, out crossed with *Canton S* for 10 generations, was used as control. UAS-mCD8-GFP, *slowpoke1* [43] were obtained from the Bloomington Drosophila Stock Centre, *pain1*(EP(2)2452), *pain2* (EP(2)2621), *pain3*(EP(2)2251), and *pain4*(EP(2)2462) from Szeged collection. All are generated by EP insertions, two of them (*pain1* and *pain3*) having UAS motifs and basal promotor inserted in the orientation which leads to endogenous *pain* gene ectopic expression when crossed to Gal4 expressing line (see scheme in Figure 2E). UAS-dsRNA lines were either obtained from VDRC, or previously described (Ork1, [11]) or constructed in the lab (Ih and NDAE1, see below). Gal4 drivers are NP1029-Gal4 [15] and dHand-Gal4 (from A. Paululat). UAS>*dTRPAFL* (from P.A. Garrity, [44]) and UAS>*nanchungFL* and UAS>*waterwitchFL* (from J. Welsh, [45]).

### UAS-dsRNA>*Ih^(a)^ and >Ndae1^(b)^* constructs

A 302***^(a)^*** and 524***^(b)^*** bp fragment from the coding sequence of the *Ork1* gene (CG8585***^(a)^*** and CG42253***^(b)^***) was PCR amplified from genomic DNA (primer sequences available upon request) and cloned in the pWIZ vector digested by Avr II (pWIZ/*Ih* ***^(a)^*** and *NDAE* ***^(b)^*** {1}). A pWIZ/*Ih* ***^(a)^*** and *NDAE* ***^(b)^*** {1} clone was then digested by Nhe I and the 302***^(a)^*** and 524***^(b)^*** bp amplified product was cloned in an inverted orientation to yield to the pWIZ/*Ih* ***^(a)^*** and *NDAE* ***^(b)^*** {1,2}.

### UAS>*painlessFL* constructs

The full length *painless* cDNA from BDGP gold collection (RE03641) was excised from pFCLI by KpnI and NotI digestion and inserted into KpnI/NotI-cut pUAST-attb transformation vector [46].

### *In Situ* hybridizations

*In situ* hybridizations on dissected larval individuals were performed as previously described [15].

### Heart arrests examination and heart rate measurements

Larvae were maintained at 18°C, 21°C or 29°C for gain of function, mutant or loss of function experiments respectively. Larvae were kept at 25°C (measurements temperature) 30 minutes before each experiment. For heart arrests analysis following larval crawling, individuals were simply mounted on glass slides using self-adhesive tape. For heart rate measurement, larvae were anaesthetized with FlyNap (Carolina Biological, Burlington, NC, USA) once mounted on double-faced tape. Movies caption, data acquisition and analyses were performed as previously described [11]. For each individual, 11 second-video sequences were acquired to determine the heart rate and to measure the maximal diastole and maximal systole diameters in the A6 segment. Contractility was determined by (Diastole max – Systole max)/Diastole max.

### Physiological tests

Larvae were carefully semi-dissected in Schneider medium (GIBCO, Invitrogen) at 25°C (the room temperature) and preparations were kept 5 minutes in Schneider medium before each experiments.

#### Pressure test

a glass pipette was fire-polished and mounted on a micromanipulator to precisely control its displacement. Pressure was applied to the cardiomyocytes of the posterior A6 segment. Heart beating was recorded with a LEICA MZ12 binocular and a DXC-107AP color video camera (Sony). Data acquisition was performed with the PCTV vision software (Pinnacle Systems).

#### Temperature test

heart beating was recorded (as indicated above) in Schneider medium at 25°C. Schneider medium was subsequently substituted by heated medium at 36°, 46° and 50°C (as indicated in Results) and new video movies were made. Temperature is measured in the bath each 15 sec to follow the decrease over time.

#### Hypoosmotic shock

Schneider medium was substituted by a standard solution containing in mM: 5.4 CaCl_2_, 25 KCl, 15 MgCl_2_, 70 NaCl, 4.3 NaHCO_3_, 80 D-glucose, 5 trehalose, 5 L-glutamine, 10 Hepes-NaOH buffer, pH = 6.9 and osmolarity 350 mOsm. One minute is sufficient to recover the basal beating of the cardiac tube after medium substitution. Heart beating was recorded with a Leica DMRX-A2 straight light microscope and a HCX APO 10×0.30 objective, with a high-resolution video camera (CoolSNAP HQ Monochrome, Roper Scientific, Inc). The standard solution was subsequently replaced by the hypoosmotic solution with the same composition as the standard medium except for NaCl (46 mM) and D-glucose (11 mM) leading to an osmolarity of 250 mOsm. Heart beating was recorded as described above. Data acquisition was performed with the Metamorph/Metafluor software (Universal Imaging, West Chester, PA).

#### M-mode

The movement of the heart was analyzed using a previously described software developed under Matlab application [47]. The software tracks movement of the heart edges using both changes in average light intensity of each frame and changes in the intensity in each individual pixel from frame to frame. M-modes were created by the software by electronically “cutting” out a single specified vertical row of pixels that span the heart tube from every frame of the movie, and aligning them horizontally. The M-modes describe the vertical movement of the heart walls over time.

### Action Potentials recordings

APs recordings on dissected larval individuals were performed as previously described [11].

### Statistical analysis

Data are reported as mean ± SD. The significance between groups of data was assessed by Student's t test (for unpaired samples) and one-way analysis of variance (ANOVA test) when three or more groups were compared. Results were considered significant with p less than 0.05 (*p<0.005, **p<0.0005, ***p<0.0001).

## Supporting Information

Table S1 Genetic screen for genes implicated in cardiac activity. The genes analyzed are grouped according to the functions of the proteins they encode. Cardiac expression of tested genes was verified by RT-PCR with RNA extracted from third instar larvae dissected cardiac tubes. Heart rate variation in knock-downed larvae was measured in vivo in GFP-expressing hearts of anaesthetized third instar larvae. Mechanosensitivity was scored by direct observation in non anaesthetized, immobilized third instar larvae. Heart rate are compared to wild-type controls (***p<0.0001).
(0.06 MB DOC)

Video S1 Cardiac tube expression of a membrane bound GFP driven by the NP1029-Gal4 driver in wild-type larva. Larva is stuck by its ventral side on double-faced tape and exhibit strong muscular contractions when attempting to crawl. Cardiac transient arrest is observed during these muscular contractions. (Posterior is right).
(9.64 MB AVI)

Video S2 Cardiac tube expression of membrane bound GFP driven by the NP1029-Gal4 driver on anaesthetized wild-type larva in which all body wall muscle movements are abolished. The heart beats regularly and no pauses in cardiac activity are observed. (Posterior is right).
(8.90 MB AVI)

Video S3 Cardiac tube expression of membrane bound GFP driven by the NP1029-Gal4 driver in wild-type larva. Cardiac transient arrest is observed during the general contraction of the larva. (Posterior is right).
(1.88 MB AVI)

Video S4 Cardiac tube expression of membrane bound GFP driven by the NP1029-Gal4 driver in larva in which *pain* is specifically inactivated by RNAi in the cardiac tube. A clear absence of cardiac pauses is observed during the general contraction of the larva. (Posterior is right).
(4.11 MB AVI)

Video S5 Cardiac tube expression of membrane bound GFP driven by NP1029-Gal4 in *Pain4* mutant larva. The larva displayed no cardiac arrests when attempting to crawl. (Posterior is right).
(3.92 MB AVI)

Video S6 Cardiac tube expression of membrane bound GFP driven by NP1029-Gal4 in *pain3* mutant larva with cardiac-specific re-expression of *pain* cDNA using the UAS/Gal4 system. The mutant phenotype is rescued and cardiac arrests are observed during the general contraction of the larva. (Posterior is right).
(1.56 MB AVI)

Video S7 Non expressing-GFP cardiac tube in *Pain4* mutant larva with cardiac-specific expression of the *pain* cDNA using the UAS/Gal4 system. The mutant phenotype is rescued and cardiac arrests are observed during the general contraction of the larva. Note that the absence of GFP in the cardiac tube strongly decreases the quality of the recording. (Posterior is right).
(1.96 MB AVI)
