# Supplementary material for: Response to Mechanical Stress Is Mediated by the TRPA Channel Painless in the Drosophila Heart
Source: PLoS Genet. 2010 Sep 2;6(9):e1001088. doi: 10.1371/journal.pgen.1001088 (PMC2932686; doi:10.1371/journal.pgen.1001088)
Supplement: Table S1 — Genetic screen for genes implicated in cardiac activity. The genes analyzed are grouped according to the functions of the proteins they encode. Cardiac expression of tested genes was verified by RT-PCR with RNA extracted from third instar larvae dissected cardiac tubes. Heart rate variation in knock-downed larvae was measured in vivo in GFP-expressing hearts of anaesthetized third instar larvae. Mechanosensitivity was scored by direct observation in non anaesthetized, immobilized third instar larvae. Heart rate are compared to wild-type controls (***p<0.0001). (0.06 MB DOC) [file pgen.1001088.s001.doc]

| **Table S1. Genetic screen for genes implicated in cardiac activity** | | | | | | | |
| --- | --- | --- | --- | --- | --- | --- | --- |
| Protein Familly | UAS-dsRNA>*gene X* | CG | RNAi source | Transformant ID | Cardiac expression | Heart rate variation (%) | Mechanosensitivity |
| Trp channel | TrpM | 4541 | vdrc | 30609 | + | no | ok |
|  | TrpM | 4541 | vdrc | 30610 |  | no | ok |
|  | Painless | 15860 | vdrc | 39477 | **+** | **17***** | **no** |
|  | Painless | 15860 | vdrc | 39478 |  | **18***** | **no** |
|  | Nanchung | 5842 | vdrc | 5260 | + | no | ok |
|  | Waterwitch | 31284 |  | 1.3 | + | no | ok |
|  | TrpA1 | 5751 | vdrc | 37249 | - | no | ok |
|  | TrpA1 | 5751 | vdrc | 37250 |  | no | ok |
|  | Pkd2 | 6504 | vdrc | 6940 | + | no | ok |
|  | Pkd2 | 6504 | vdrc | 6941 |  | no | ok |
| Calcium-activated potassium channel | Slowpoke | 10693 | vdrc | 6722 | + | no | ok |
|  | Slowpoke | 10693 | vdrc | 6723 |  | no | ok |
|  | SK | 10706 | vdrc | 24601 | + | **-19***** | ok |
| Potassium/sodium channel | Ih | 8585 | team | 7.1 | + | no | ok |
|  | Ih | 8585 | team | 7.3 |  | no | ok |
|  | Ih | 8585 | team | 12.1 |  | no | ok |
| Two-pore potassium channel | Ork1 | 1615 | team | 4.1 | + | **20***** | ok |
|  | Ork1 | 1615 | team | 6.3 |  | **25***** | ok |
|  | Ork1 | 1615 | team | 7.1 |  | **25***** | ok |
| Calcium channel | Alpha 1D | 4894 | vdrc | 51491 | + | **arrest** | undetermined |
|  | Ca-beta | 42403 | vdrc | 27581 | + | no | ok |
| IP3 receptor | IP_3_ | 34359 | vdrc | 19151 | + | no | ok |
| Calmodulin | Calmodulin | 8472 | vdrc | 28242 | undetermined | no | ok |
|  | Calmodulin | 8472 | vdrc | 102004 |  | **arrest** | undetermined |
| Chlorure channel | Axs-like | 15270 | vdrc | 3830 | - | **24***** | ok |
| Na+-driven anion exchanger 1 | Ndae1 | 42253 | team | 55.26 | + | no | ok |
|  | Ndae1 | 42253 | vdrc | 967 |  | no | ok |
